# Subclinical Measures of Peripheral Atherosclerosis and the Risk of New‐Onset Atrial Fibrillation in the General Population: the Rotterdam Study

**DOI:** 10.1161/JAHA.121.023967

**Published:** 2021-12-31

**Authors:** Sven Geurts, Cathrine Brunborg, Grigorios Papageorgiou, M. Arfan Ikram, Maryam Kavousi

**Affiliations:** ^1^ Department of Epidemiology Erasmus MC, University Medical Center Rotterdam Rotterdam The Netherlands; ^2^ Oslo Centre for Biostatistics and Epidemiology Research Support Services, Oslo University Hospital Oslo Norway; ^3^ Department of Biostatistics Erasmus MC, University Medical Center Rotterdam Rotterdam The Netherlands

**Keywords:** atrial fibrillation, epidemiology, peripheral atherosclerosis, risk factors, sex‐differences, Atrial Fibrillation, Epidemiology, Risk Factors, Atherosclerosis, Women

## Abstract

**Background:**

Limited population‐based data on the (sex‐specific) link between subclinical measures of peripheral atherosclerosis and new‐onset atrial fibrillation (AF) exist.

**Methods and Results:**

Subclinical measures of peripheral atherosclerosis including carotid intima‐media thickness (cIMT), carotid plaque, and ankle‐brachial index (ABI) were assessed at baseline and follow‐up examinations. A total of 12 840 participants free of AF at baseline from the population‐based Rotterdam Study were included. Cox proportional hazards models and joint models, adjusted for cardiovascular risk factors, were used to determine the associations between baseline and longitudinal measures of cIMT, carotid plaque, and ABI with new‐onset AF. During a median follow‐up of 9.2 years, 1360 incident AF cases occurred among 12 840 participants (mean age 65.2 years, 58.3% women). Higher baseline cIMT (fully‐adjusted hazard ratio [HR], 95% CI, 1.81, 1.21–2.71; *P*=0.0042), presence of carotid plaque (fully‐adjusted HR, 95% CI, 1.19, 1.04–1.35; *P*=0.0089), lower ABI (fully‐adjusted HR, 95% CI, 1.57, 1.14–2.18; *P*=0.0061) and longitudinal measures of higher cIMT (fully‐adjusted HR, 95% CI, 2.14, 1.38–3.29; *P*=0.0021), presence of carotid plaque (fully‐adjusted HR, 95% CI, 1.61, 1.12–2.43; *P*=0.0112), and lower ABI (fully‐adjusted HR, 95% CI, 4.43, 1.83–10.49; *P*=0.0007) showed significant associations with new‐onset AF in the general population. Sex‐stratified analyses showed that the associations for cIMT, carotid plaque, and ABI were mostly prominent among women.

**Conclusions:**

Baseline and longitudinal subclinical measures of peripheral atherosclerosis (carotid atherosclerosis, and lower extremity peripheral atherosclerosis) were significantly associated with an increased risk of new‐onset AF, especially among women.

**Registration:**

URL: https://www.trialregister.nl, https://www.apps.who.int/trialsearch/; Unique identifier: NL6645/NTR6831.

**Nonstandard Abbreviations and Acronyms**
DM: diabetes mellitus
ESC: European Society of Cardiology
MEANS: Modular ECG Analysis System
n: number

**Clinical Perspective**

**What Is New?**

- Limited population‐based data on the (sex‐specific) link between subclinical measures of peripheral atherosclerosis (carotid intima‐media thickness, carotid plaque, and ankle‐brachial index) and new‐onset atrial fibrillation exist.
- In this large population‐based cohort study, we found that baseline and longitudinal subclinical measures of peripheral atherosclerosis were significantly associated with new‐onset atrial fibrillation; sex‐stratified analyses showed that the associations for carotid intima‐media thickness, carotid plaque, and ankle‐brachial index were mostly prominent among women.
- Our findings suggest that subclinical measures of peripheral atherosclerosis may be independent risk factors for atrial fibrillation pathogenesis.

**What Are the Clinical Implications?**

- Our findings imply that treatment to reduce subclinical peripheral atherosclerosis might carry a potential for prevention of atrial fibrillation in the general population, especially among women, but future experimental studies are warranted to confirm our findings.

Atrial fibrillation (AF) is the most prevalent cardiac arrhythmia.^1^, ^2^ Parallel to the aging of the population, the prevalence of AF is expected to increase steeply in the coming years.^1^, ^2^, ^3^ Despite improvements in the management of patients with AF, it still confers a large morbidity and mortality risk.^1^, ^2^, ^4^ Notably, recent evidence points towards sex‐differences in the pathophysiology and prognosis of AF.^5^, ^6^ Women with AF are older at diagnosis, have a higher prevalence of hypertension and valvular heart disease, and have an increased risk of stroke, myocardial infarction, and mortality in comparison with men.^5^

Atherosclerosis of the peripheral vasculature is a largely prevalent condition in the general population that is associated with increased risk of morbidity and mortality.^7^ Peripheral atherosclerosis and AF share common major risk factors.^8^ Previous reports have suggested a relationship between peripheral atherosclerosis and AF mainly based on subgroup or post hoc analyses of various patient studies.^8^ Few population‐based studies have shown associations between subclinical measures of peripheral atherosclerosis; carotid intima‐media thickness (cIMT),^9^, ^10^, ^11^, ^12^ carotid plaque,^9^, ^10^ and ankle‐brachial index (ABI)^7^, ^13^, ^14^ with increased risk of new‐onset AF. To date, limited data on the link between longitudinal measures of peripheral atherosclerosis with new‐onset AF in the general population exist. Moreover, comprehensive assessment of the sex‐specific association between the 2 conditions is sparse.

We thus aimed to investigate the associations between baseline and longitudinal measures of subclinical peripheral atherosclerosis including cIMT, carotid plaque, and ABI with the risk of new‐onset AF among participants from the large population‐based Rotterdam Study. Additionally, we sought to evaluate sex‐differences with regard to the association between subclinical peripheral atherosclerosis and new‐onset AF.

## METHODS

Data can be obtained upon request. Requests should be directed towards the management team of the Rotterdam Study (secretariat.epi@erasmusmc.nl), which has a protocol for approving data requests. Because of restrictions based on privacy regulations and informed consent of the participants, data cannot be made freely available in a public repository.

### Study Population

We used data from the Rotterdam Study.^15^, ^16^ The Rotterdam Study is a prospective population‐based cohort study that aims to assess the occurrence and progression of risk factors for chronic diseases in middle‐age and elderly people. During 1990–1993, all inhabitants of the Ommoord district in the city of Rotterdam in The Netherlands aged ≥55 years were invited for the study. A total of 7983 (78% of all invitees) agreed to participate (RS‐I). In 2000, the cohort was extended with 3011 participants who had become aged ≥55 years or had migrated into the research area (RS‐II). In 2006, the cohort was again extended with 3932 participants that were ≥45 years (RS‐III). The overall response rate at baseline was 72%. Participants attended follow‐up examinations every 3 to 5 years. Outcome data on morbidity and mortality were continuously collected through linkage with digital files from general practitioners in the study area.^15^, ^16^

The Rotterdam Study complies with the Declaration of Helsinki and has been approved by the Medical Ethics Committee of the Erasmus MC (registration number MEC 02.1015) and by the Dutch Ministry of Health, Welfare and Sport (Population Screening Act WBO, license number 1071272‐159521‐PG). The Rotterdam Study Personal Registration Data collection is filed with the Erasmus MC Data Protection Officer under registration number EMC1712001. The Rotterdam Study has been entered into the Netherlands National Trial Register (www.trialregister.nl/trials) and into the World Health Organization International Clinical Trials Registry Platform (https://apps.who.int/trialsearch/) under shared catalogue number NL6645/NTR6831. All participants provided written informed consent to participate, prior to inclusion, in the study and to have their information obtained from treating physicians.

For the current study, we included participants at study entry of the 3 recruitment waves. Participants with prevalent AF at baseline (n=559), no informed consent for follow‐up data collection (n=305), no follow‐up time (n=6) or no subclinical measures of peripheral atherosclerosis (n=1216), mainly due to logistic reasons, were excluded. Among the 12 840 participants free of AF at baseline who were included, 11 971 had at least 1 available measurement for cIMT, 11 947 had at least 1 available measurement for carotid plaque, and 8532 had at least 1 available measurement for ABI; 6832 participants had 2 measurements for cIMT, 6311 had 2 measurements for carotid plaque, and 3123 had 2 measurements for ABI; 1075 participants had 3 measurements for cIMT, and 961 had 3 measurements for carotid plaque.

### Subclinical Measures of Peripheral Atherosclerosis

Participants were assessed for cIMT, carotid plaque, and ABI at baseline and follow‐up examinations. Measurement of cIMT was performed with ultrasonography of both the left and right carotid arteries using a 7.5 MHz linear array transducer with a Duplex scanner (ATL UltraMark IV, Advanced Technology Laboratories, Bethel, Washington). The cIMT was calculated as the mean from the near and far walls measurements of both the left and right carotid arteries.^17^ Carotid plaque was assessed by examining the ultrasonographic images at common, internal, and bifurcation sites of the carotid artery for presence of atherosclerotic lesions. Presence of carotid plaque was defined as a focal widening relative to adjacent segments with protrusion into the lumen composed of only calcified deposits or combination of calcified and non‐calcified material.^17^

ABI was defined as the ratio of the systolic blood pressure at the ankle to the systolic blood pressure at the arm and was calculated for each leg. Ankle systolic blood pressure was measured in both right and left posterior tibial arteries using a Doppler ultrasound transducer with random‐zero sphygmomanometer with the patients in supine position. The lowest ABI in either leg was used in the analyses. Peripheral artery disease was defined as ABI **≤**0.9. Values of ABI >1.4 were excluded, because high ABI may represent a different underlying pathology related to calcified, non‐compressible arterial vessels.^18^, ^19^

### Assessment of AF

AF was defined in accordance with the European Society of Cardiology (ESC) guidelines.^4^ Methods on event adjudication for prevalent and incident AF have been described previously.^15^ In short, to assess AF at baseline and follow‐up examinations a 10‐second 12‐lead ECG was used with an ACTA Gnosis IV ECG recorder (Esaote; Biomedica, Florence, Italy). The ECG records were stored digitally and analyzed with the Modular ECG Analysis system (MEANS).^20^, ^21^, ^22^ Subsequently, 2 research physicians, blinded to the MEANS diagnosis, validated the diagnosis of AF. In case of disagreement a cardiologist was consulted.^3^, ^9^ Additional follow‐up data were obtained from medical files of participating general practitioners, hospitals, outpatient clinics, national registration of all hospitals discharge diagnoses, and follow‐up examinations at the research center. The date of incident AF was defined as the date of the first occurrence of symptoms suggestive of AF with subsequent ECG verification obtained from the medical records. Participants were followed from the date of enrollment in the Rotterdam Study until the date of onset of AF, date of death, loss to follow‐up, or to January 1, 2014, whichever occurred first.

### Assessment of Cardiovascular Risk Factors

The cardiovascular risk factors included in the study were body mass index, total cholesterol, high‐density lipoprotein cholesterol, hypertension, smoking status, history of diabetes, history of coronary heart disease (CHD), history of heart failure, left ventricular hypertrophy on the ECG, use of cardiac medication, and use of lipid lowering medication. Methods for measurements of cardiovascular risk factors are explained in details in Data S1.

### Statistical Analysis

Participant characteristics at study entry are presented as mean with SD or number (n) with percentages as appropriate. Differences between men and women were examined by Student's *t*‐test for continuous variables and Chi‐Square Test for categorical variables. The distribution of cIMT, and ABI were normal. Therefore, no transformation was needed.

Competing risk analyses were performed using Cox proportional hazards models to investigate the relationship between subclinical measures of peripheral atherosclerosis at baseline (cIMT, carotid plaque, and ABI) with incident AF. Cause‐specific hazard ratios (HRs) with 95%CIs were calculated to quantify the associations. For continuous exposure variables an examination of the shape of relation with incident AF was performed using natural cubic splines. No deviation from linearity was found. The proportional hazard assumptions were assessed using Schoenfeld residuals and were found to be satisfied.

Further, to investigate the associations between repeated measures of peripheral atherosclerosis over time with the risk of incident AF, joint models for longitudinal and time to event data were used. First, an appropriate, for each outcome, mixed effects model was used to analyze the longitudinal measures of peripheral atherosclerosis over time and to account for the correlation of repeated measurements. More specifically, for cIMT a linear mixed effects model was used with random intercepts and random slopes, including a linear effect of time. Moreover, for carotid plaque a logistic mixed effects model was used with random intercepts and random slopes, including a linear effect of time. Finally, for ABI a linear mixed effects model was used with random intercepts and random slopes, including a linear effect of time. Time was measured in years after baseline and cardiovascular risk factors/covariates were treated as fixed effects in all models. Likelihood Ratio Tests were used to assess whether random slopes could be dropped from the model. Due to the low number of repeated measurements per individual (range, 1–3), non‐linear functions of time using splines were not used. Next, the estimated subject‐specific trajectories from the mixed effects models were included in the Cox models as time‐varying covariates under the joint modeling framework.^23^

Analyses were performed in the total study population and for men and women separately. In addition, for the Cox proportional hazards models we tested the interaction of sex using the Likelihood Ratio Test with the individual subclinical measures of peripheral atherosclerosis in the total study population in model 1 and model 2. Similarly, we also reported the *P* values of sex interaction from the joint model in both models. All models (mixed‐ and survival models) were adjusted for age, sex (if applicable), and cohort (model 1) and additionally for cardiovascular risk factors including body mass index, total cholesterol, high‐density lipoprotein cholesterol, hypertension, smoking status, history of diabetes, history of CHD, history of heart failure, left ventricular hypertrophy on the ECG, use of cardiac medication, and use of lipid lowering medication (model 2). Missing baseline covariate values were imputed under the assumption of missing at random and were imputed using predictive mean matching (“pmm”), binary logistic regression (“logreg”), and a proportional odds model (“polyr”) for continuous, binary, and ordered categorical covariates, respectively, from the “mice” package in R.^24^ For imputation all available data were used to generate 1 imputed dataset. Missing values: body mass index (1.9%), total cholesterol (2.5%), high‐density lipoprotein cholesterol (2.5%), systolic blood pressure (0.6%), diastolic blood pressure (0.6%), smoking status (1.4%), history of CHD (3.5%), history of heart failure (0.2%), left ventricular hypertrophy on the ECG (18.7%), use of cardiac medication (0.7%), use of antihypertensive medication (0.7%), and use of lipid lowering medication (0.7%).

Multiple sensitivity analyses were performed. We compared the analyses with imputed data and complete case analyses. Moreover, we reran our analyses after excluding participants with prevalent and incident CHD (prior to incident AF) to evaluate if this would attenuate the observed associations. Finally, we also calculated the cause‐specific HRs for mortality to evaluate the competing risk of mortality with incident AF.

Statistical significance was considered at 2‐tailed *P*<0.05 or for the Bayesian joint models, a tail probability of <0.05. The data management was done using IBM SPSS Statistics version 25.0 for Windows (IBM Corp, Armonk, New York). The statistical analyses were performed using the R package “JMbayes2”^25^ in R software (R 4.0.2; R Foundation for Statistical Computing, Vienna, Austria).^26^

## RESULTS

A total of 12 840 participants free of AF at baseline, 5359 men (41.7%) and 7481 women (58.3%), were eligible for the analyses. The baseline characteristics for the total study population and for the study population stratified by sex are depicted in Table 1. The mean age of the total study population was 65.2±9.8 years and 58.3% were women.

**Table 1:** Baseline Characteristics of the Total Study Population and Stratified by Sex

Baseline characteristics*	Total study population (n=12 840)	Men (n=5359)	Women (n=7481)	*P* value^†^
Age, y	65.2±9.8	64.4±9.1	65.8±10.3	<0.001
Women, n (%)	7481 (58.3)	NA	7481 (100)	NA
Body mass index, kg/m^2^	26.9±4.1	26.6±3.6	27.2±4.5	<0.001
Total cholesterol, mmol/L^‡^	6.1±1.2	5.8±1.2	6.3±1.2	<0.001
High‐density lipoprotein cholesterol, mmol/L^‡^	1.4±0.4	1.2±0.3	1.5±0.4	<0.001
Systolic blood pressure, mm Hg	139.0±21.7	139.8±20.9	138.5±22.3	0.001
Diastolic blood pressure, mm Hg	77.6±12.0	78.8±12.0	76.7±11.9	<0.001
Hypertension, n (%)	7628 (59.4)	3218 (60.0)	4410 (58.9)	0.211
Smoking status, n (%)				<0.001
Never	4114 (32.5)	751 (14.1)	3363 (45.7)	
Former	5514 (43.5)	3010 (56.7)	2504 (34.0)	
Current	3038 (24.0)	1550 (29.2)	1488 (20.2)	
History of diabetes, n (%)	1334 (10.4)	632 (11.8)	702 (9.4)	<0.001
History of coronary heart disease, n (%)	804 (6.5)	572 (11.0)	232 (3.2)	<0.001
History of heart failure, n (%)	220 (1.7)	84 (1.6)	136 (1.8)	0.278
Left ventricular hypertrophy, n (%)	683 (6.5)	394 (9.0)	289 (4.8)	<0.001
Cardiac medication, n (%)	810 (6.4)	366 (6.9)	444 (6.0)	0.039
Antihypertensive medication, n (%)	779 (6.1)	329 (6.2)	450 (6.1)	0.762
Lipid lowering medication, n (%)	1376 (10.8)	658 (12.4)	718 (9.7)	<0.001
Carotid intima‐media thickness, mm^§^	0.82±0.15	0.85±0.15	0.80±0.14	<0.001
Carotid plaque, n (%)^‖^	7918 (66.3)	3660 (72.7)	4258 (61.6)	<0.001
Ankle‐brachial index^¶^	1.05±0.19	1.08±0.19	1.04±0.19	<0.001
Peripheral artery disease, n (%)^¶^	1366 (16.0)	507 (14.4)	859 (17.2)	0.001

Values are shown before imputation and therefore not always add up to 100%.

* Values are mean (SD) for continuous variables or number (percentages) for categorical variables.

^†^ Statistical significance for continuous data was tested using the Student's *t‐*test and for categorical data was tested using the Chi‐square Test.

^‡^ SI conversion factor: to convert cholesterol to mg/dL divide values by 0.0259.

^§^ The baseline population for carotid intima‐media thickness measurements included 5048 men and 6923 women.

^‖^ The baseline population for carotid plaque measurements included 5033 men and 6914 women.

^¶^ The baseline population for ankle‐brachial index measurements included 3525 men and 5007 women. Peripheral artery disease was defined as ankle‐brachial index ≤0.9.

During a median follow‐up time of 9.2 years (interquartile range, 6.1–14.3), 1360 incident AF cases (10.6%) (640 in men and 720 in women) and 4348 mortality cases (33.9%) 1879 in men and 2469 in women) occurred. The incidence rate of AF was 9.8 per 1000 person‐years in the total study population (11.8 per 1000 person‐years in men, 8.6 per 1000 person‐years in women) and the incidence rate of mortality was 31.5 per 1000 person‐years in the total study population (34.6 per 1000 person‐years in men, 29.5 per 1000 person‐years in women).

The Cox proportional hazards analyses showed significant associations between higher baseline cIMT (HR, per 1 unit increase, 95% CI, 2.98, 2.01–4.42; *P*=5.22×10^−08^), presence of carotid plaque (HR, per 1 unit increase in the probability, 95% CI, 1.30, 1.15–1.48; *P*=4.06×10^−05^), and lower ABI (HR, per 1 unit decrease, 95% CI, 2.11, 1.55–2.87; *P*=2.32×10^−06^) with an increased risk of new‐onset AF in the total study population in model 1. For the Cox proportional hazards models, the results of the sex interaction testing for cIMT, carotid plaque, and ABI in the total study population were *P*=0.0047, *P*=0.5534, *P*=0.7621, respectively. After adjusting for additional cardiovascular risk factors in model 2, the effect estimates attenuated, but higher cIMT (HR, per 1 unit increase, 95% CI, 1.81, 1.21–2.71; *P*=0.0042), presence of carotid plaque (HR, per 1 unit increase in the probability, 95% CI, 1.19, 1.04–1.35; *P*=0.0089), and lower ABI (HR, per 1 unit decrease, 95% CI, 1.57, 1.14–2.18; *P*=0.0061) remained significantly associated with the risk of new‐onset AF in the total study population (Tables 2 and 3). In model 2, the results of the sex interaction testing for cIMT, carotid plaque, and ABI in the total study population were *P*=0.0011, *P*=0.2535, *P*=0.9317, respectively. Additionally, analyses of quartiles of cIMT (HR, 95% CI, 1.31, 1.10–1.57; *P*=0.0030, for the highest versus lowest quartile) and categories of ABI (HR, 95% CI, 1.21, 1.02–1.42; *P*=0.0249 for the lowest (≤0.90) versus highest (1.00–1.40) category) showed significant graded associations between increased quartiles of cIMT and lower categories of ABI with incident AF in model 2 (Tables 2 and 3).

**Table 2:** Association Between Baseline and Longitudinal Measures of Carotid Intima‐Media Thickness and Carotid Plaque With the Risk of New‐Onset Atrial Fibrillation in the Total Study Population and Stratified by Sex

	Total study population		Men		Women	
	Cause‐specific HR (95% CI)					
	Model 1*	Model 2^†^	Model 1*	Model 2^†^	Model 1*	Model 2^†^
Cox proportional hazards models^‡^						
cIMT^‖^	2.98 (2.01–4.42), *P*=5.22×10^−08^	1.81 (1.21–2.71), *P*=0.0042	1.70 (0.97–2.99), *P*=0.0642	1.00 (0.56–1.80), *P*=0.9989	5.26 (3.05–9.09), *P*=2.66×10^−09^	3.32 (1.90–5.80), *P*=2.49×10^−05^
Carotid plaque^‖^	1.30 (1.15–1.48), *P*=4.06×10^−05^	1.19 (1.04–1.35), *P*=0.0089	1.25 (1.03–1.50), *P*=0.0218	1.10 (0.91–1.33), *P*=0.3399	1.35 (1.14–1.59), *P*=0.0006	1.27 (1.07–1.51), *P*=0.0065
cIMT, quartiles^‖^						
Q1^¶^	1 (ref)	1 (ref)	1 (ref)	1 (ref)	1 (ref)	1 (ref)
Q2^¶^	1.16 (0.97–1.38), *P*=0.1021	1.07 (0.90–1.27), *P*=0.4755	1.15 (0.90–1.46), *P*=0.2571	1.06 (0.83–1.34), *P*=0.6633	1.21 (0.95–1.55), *P*=0.1210	1.11 (0.87–1.42), *P*=0.4131
Q3^¶^	1.32 (1.11–1.57), *P*=0.0019	1.18 (0.99–1.40), *P*=0.0672	1.21 (0.95–1.54), *P*=0.1179	1.05 (0.82–1.34), *P*=0.6832	1.33 (1.04–1.71), *P*=0.0218	1.21 (0.95–1.55), *P*=0.1259
Q4^¶^	1.58 (1.33–1.89), *P*=3.43×10^−07^	1.31 (1.10–1.57), *P*=0.0030	1.31 (1.03–1.68), *P*=0.0301	1.07 (0.83–1.38), *P*=0.6104	1.77 (1.38–2.27), *P*=6.96×10^−06^	1.50 (1.16–1.92), *P*=0.0016
Joint models^§^						
cIMT^‖^	3.38 (2.20–5.23), *P*<0.0001	2.14 (1.38–3.29), *P*=0.0021	1.87 (1.01–3.47), *P*=0.0449	1.12 (0.58–2.22), *P*=0.7460	6.59 (3.58–12.18), *P*<0.0001	4.31 (2.23–8.12), *P*<0.0001
Carotid plaque^‖^	2.05 (1.42–3.03), *P*=0.0028	1.61 (1.12–2.43), *P*=0.0112	1.86 (1.12–3.11), *P*=0.0049	1.23 (0.78–1.96), *P*=0.3796	2.11 (1.38–3.33), *P*=<0.0001	1.82 (1.17–2.81), *P*=0.0084

cIMT, carotid intima‐media thickness; HR, hazard ratio; and Q, quartiles.

*Adjusted for age, sex (if applicable), and cohort.

^†^Adjusted for age, sex (if applicable), cohort, body mass index, total cholesterol, high‐density lipoprotein cholesterol, hypertension, smoking status, history of diabetes, history of coronary heart disease, history of heart failure, left ventricular hypertrophy on the ECG, use of cardiac medication, and use of lipid lowering medication.

Association between ^‡^ baseline carotid intima‐media thickness and ^§^ longitudinal measures of carotid intima‐media thickness, and carotid plaque for up to 3 repeated measurements during follow‐up with incident atrial fibrillation, assessed by ^‡^ Cox proportional hazards models and ^§^ joint models.

^‖^ Hazard ratios represent 1 unit increase in carotid intima‐media thickness, and 1 unit increase in the probability of carotid plaque with the risk of new‐onset atrial fibrillation.

^¶^ Quartiles in the total study population were Q1: ≤0.72 mm, Q2: 0.73 to 0.80 mm, Q3: 0.81 to 0.90 mm, Q4: ≥0.91 mm. Quartiles in men were Q1: ≤0.74 mm, Q2: 0.75 to 0.83 mm, Q3: 0.84 to 0.94 mm, Q4: ≥0.95 mm. Quartiles in women were Q1: ≤0.70 mm, Q2: 0.71 to 0.78 mm, Q3: 0.79 to 0.88 mm, Q4: ≥0.89 mm.

**Table 3:** Association Between Baseline and Longitudinal Measures of Ankle‐Brachial Index With the Risk of New‐Onset Atrial Fibrillation in the Total Study Population and Stratified by Sex

	Total study population		Men		Women	
	Cause‐specific HR (95% CI)					
	Model 1*	Model 2^†^	Model 1*	Model 2^†^	Model 1*	Model 2^†^
Cox proportional hazards models^‡^						
ABI^‖^	2.11 (1.55–2.87), *P*=2.32×10^−06^	1.57 (1.14–2.18), *P*=0.0061	2.29 (1.47–3.57), *P*=0.0003	1.62 (1.01–2.59), *P*=0.0447	1.95 (1.27–3.00), *P*=0.0023	1.53 (0.97–2.39), *P*=0.0654
ABI, categories^‖^						
≤0.90	1.34 (1.14–1.57), *P*=0.0004	1.21 (1.02–1.42), *P*=0.0249	1.45 (1.14–1.85), *P*=0.0029	1.27 (0.99–1.63), *P*=0.0625	1.25 (1.01–1.56), *P*=0.0397	1.17 (0.94–1.45), *P*=0.1707
0.91–0.99	1.29 (1.09–1.53), *P*=0.0037	1.17 (0.99–1.40), *P*=0.0696	1.40 (1.08–1.83), *P*=0.0113	1.25 (0.96–1.63), *P*=0.1011	1.21 (0.97–1.52), *P*=0.0961	1.11 (0.88–1.39), *P*=0.3757
1.00–1.40	1 (ref)	1 (ref)	1 (ref)	1 (ref)	1 (ref)	1 (ref)
Joint models^§^						
ABI^‖^	7.53 (3.65–16.10), *P*<0.0001	4.43 (1.83–10.49), *P*=0.0007	6.53 (2.47–19.01), *P*<0.0001	3.72 (1.20–11.95), *P*=0.0225	7.84 (2.61–22.07), *P*<0.0001	5.03 (1.61–16.80), *P*=0.0042

ABI indicates ankle‐brachial index;and HR, hazard ratio.

*Adjusted for age, sex (if applicable), and cohort.

^†^Adjusted for age, sex (if applicable), cohort, body mass index, total cholesterol, high‐density lipoprotein cholesterol, hypertension, smoking status, history of diabetes, history of coronary heart disease, history of heart failure, left ventricular hypertrophy on the ECG, use of cardiac medication, and use of lipid lowering medication.

Association between ^‡^ baseline ankle‐brachial index and ^§^ longitudinal measures of ankle‐brachial index for up to 2 repeated measurements during follow‐up with incident atrial fibrillation, assessed by ^‡^ Cox proportional hazards models and ^§^ joint models.

^‖^ Hazard ratios represent 1 unit decrease in ankle‐brachial index with the risk of new‐onset atrial fibrillation.

The sex‐stratified analyses from model 2 showed that the associations for higher cIMT (HR, per 1 unit increase, 95% CI, 1.00, 0.56–1.80; *P*=0.9989), and presence of carotid plaque (HR, per 1 unit increase in the probability, 95% CI, 1.10, 0.91–1.33; *P*=0.3399) were not significant in men, while significant associations for higher cIMT (HR, per 1 unit increase, 95% CI, 3.32, 1.90–5.80; *P*=2.49×10^−05^), and presence of carotid plaque (HR, per 1 unit increase in the probability, 95% CI, 1.27, 1.07–1.51; *P*=0.0065) were found in women. Analyses of lower ABI (HR, per 1 unit decrease, 95% CI, 1.62, 1.01–2.59; *P*=0.0447) showed a significant association in men and a borderline significant association was found in women (HR, per 1 unit decrease, 95% CI, 1.53, 0.97–2.39; *P*=0.0654). Again, we observed graded associations between increased quartiles of cIMT and lower categories of ABI with incident AF in the sex‐stratified analyses (Tables 2 and 3).

The joint model analyses also showed significant associations between longitudinal measures of higher cIMT (HR, per 1 unit increase, 95% CI, 3.38, 2.20–5.23; *P*<0.0001), presence of carotid plaque (HR, per 1 unit increase in the probability, 95% CI, 2.05, 1.42–3.03; *P*=0.0028), and lower ABI (HR, per 1 unit decrease, 95% CI, 7.53, 3.65–16.10; *P*<0.0001) with an increased risk of new‐onset AF in the total study population in model 1. The *P* values of the sex interaction in model 1 in the joint model for cIMT, carotid plaque, and ABI in the total study population were *P*<0.0001, *P*=0.0027, *P*=0.0053, respectively. Adjusting for additional cardiovascular risk factors in model 2 did also attenuate the effect estimates, but higher cIMT (HR, per 1 unit increase, 95% CI, 2.14, 1.38–3.29; *P*=0.0021), presence of carotid plaque (HR, per 1 unit increase in the probability, 95% CI, 1.61, 1.12–2.43; *P*=0.0112), and lower ABI (HR, per 1 unit decrease, 95% CI, 4.43, 1.83–10.49; *P*=0.0007) remained significantly associated with the risk of new‐onset AF in the total study population (Tables 2 and 3). In model 2, the *P* values of the sex interaction in the joint model for cIMT, carotid plaque, and ABI in the total study population were *P*<0.0001, *P*=0.0200, *P*=0.0302, respectively. The corresponding HRs from the sex‐stratified joint model analyses were somewhat higher, but more or less comparable with the HRs obtained in the Cox proportional hazards analyses (Tables 2 and 3).

In our sensitivity analyses, the results after imputation did not differ substantially from the complete case analyses (Tables S1 and S2). In addition, excluding participants with prevalent and incident CHD (prior to incident AF) from our analyses did not substantially change our original results (Tables S3 and S4). Lastly, we evaluated the competing risk of mortality with incident AF and larger cIMT, presence of carotid plaque, and lower ABI were all significantly associated with mortality in both model 1 and 2 (Tables S5 and S6).

## DISCUSSION

In this large prospective population‐based cohort study, baseline and longitudinal measures of subclinical peripheral atherosclerosis were significantly associated with an increased risk of new‐onset AF in the general population. Sex‐stratified analyses indicated that the associations were mostly prominent among women. Our findings imply that treatment to reduce subclinical peripheral atherosclerosis might carry a potential for prevention of AF in the general population, especially among women.

The relationship between peripheral atherosclerosis and AF is not yet fully understood. It has been suggested that the association between peripheral atherosclerosis and AF can be in part attributable to the several shared cardiovascular risk factors.^8^, ^14^, ^27^ Common risk factors including age, sex, obesity, hypertension, and diabetes, that contribute to (peripheral) atherosclerosis do also contribute to AF development.^28^ Inflammation, endothelial dysfunction, and platelet‐mediated thrombosis have been suggested as part of the underlying mechanisms that relate peripheral atherosclerosis with AF.^8^, ^14^, ^27^, ^29^ Indeed, in our study, the associations between cIMT, carotid plaque, and ABI with incident AF attenuated after adjustment for traditional cardiovascular risk factors. However, the associations remained significant after taking into account cardiovascular risk factors. Nonetheless, it seems plausible that the combination of these aforementioned mechanisms reflects the association between peripheral atherosclerosis and AF, but further research to elucidate underlying mechanisms is warranted.

CIMT, carotid plaque, and ABI are perceived as subclinical measures of peripheral atherosclerosis and have also been linked to coronary artery disease (CAD).^8^, ^30^, ^31^, ^32^ All 3 measures provide information on the extent of atherosclerosis even during the early phases of atheroma formation.^33^ However, atherosclerosis of the carotid arteries may carry a stronger association with coronary atherosclerosis, and therefore CAD, than lower extremity atherosclerosis.^33^, ^34^ Atherosclerosis, specifically CAD, induces an increase in left ventricular filling pressure, as reflected by an enlarged left atrium. Myocardial ischemia as well induces electrical and structural remodeling of the AF substrate. These aforementioned phenomena are among the mechanisms linking atherosclerosis and CAD with AF occurrence and maintenance.^35^ Notably, besides its stronger association with CAD, cIMT has shown to be associated with pan‐vascular atherosclerosis.^36^ Notably, excluding participants with prevalent and incident CHD (prior to incident AF) did not change our original results.

Our study assessed the baseline and longitudinal measures of subclinical peripheral atherosclerosis during a long follow‐up time in relationship to new‐onset AF. Considering repeated measurements of peripheral atherosclerosis in relationship to new‐onset AF by using joint models may provide more insight and give more prognostic information over a single baseline measurement. Longitudinal measures of carotid atherosclerosis, and lower extremity peripheral atherosclerosis, during follow‐up were associated with an increased risk of incident AF, especially among women. These findings extend previous evidence by additionally reporting on repeated measurements and sex‐differences while assessing the relationship between peripheral atherosclerosis and AF.^7^, ^8^, ^9^, ^10^, ^11^, ^12^, ^13^, ^14^ We are not able to fully explain these sex‐differences, but 1 possible explanation could be due to differences in sex‐hormones. Women might benefit from the anti‐atherosclerotic characteristics of higher estrogen levels during their life span. However, this protection is rapidly lost after menopause which gives rise to various forms of cardiovascular disorders. It has been demonstrated that estrogen affects the coronary arteries, aorta, and cerebral arteries differently.^37^ We therefore hypothesize that the higher estrogen levels before menopause among women may have a larger protective effect on coronary and carotid atherosclerosis than lower extremity atherosclerosis.^37^ This might explain why cIMT and carotid plaque is only associated with incident AF in women and not in men and that ABI is associated with AF in both men and women. We further hypothesize that the association in women may be caused through a distinct pathway, other than the pathways observed in men. In particular, the effect estimates observed in men in our study attenuated the most after adjusting for traditional cardiovascular risk factors. This might imply that the pathways involved in AF pathophysiology in women might not be solely via the traditional cardiovascular risk factors. Further, previous evidence has suggested competing risk of death as a plausible explanation for these sex‐differences.^9^, ^11^ Since AF is also strongly associated with age, there may be a possibility that men die of other (cardiovascular) diseases prior to AF development and this hypothesis was supported by our competing risk analyses which showed that cIMT, carotid plaque, and ABI were significantly associated with mortality. Nevertheless, we observed a higher incidence of AF in men than women in this study.

The major strengths of this study are its population‐based nature, large sample size with detailed information on cardiovascular risk factors, meticulous adjudication of AF events, and long follow‐up time. Availability of both carotid atherosclerosis and lower extremity peripheral atherosclerosis measures allowed for direct comparison of various vascular beds in the same population. Availability of repeated measurements for subclinical peripheral atherosclerosis during follow‐up enabled us to study longitudinal measures of peripheral atherosclerosis in association with new‐onset AF. Additionally, we performed multiple sensitivity analyses including complete‐case analyses, excluding prevalent and incident CHD prior to AF events, and the use of competing risk analyses to calculate cause‐specific hazards.

There are also some limitations. We could not distinguish between paroxysmal, persistent, long‐standing persistent, and permanent AF as Holter monitoring has not been performed in this large population‐based cohort. Although, we adjusted for several cardiovascular risk factors, we cannot entirely rule out the possibility of residual confounding by other unmeasured risk factors. Finally, since our study population includes mainly elderly subjects that are mainly from European descent, our results may not be generalizable to younger populations or other ethnicities.

In this large population‐based cohort study we assessed baseline and longitudinal measures of subclinical peripheral atherosclerosis during follow‐up in relationship to new‐onset AF. We found that baseline and longitudinal measures of subclinical peripheral atherosclerosis were significantly associated with an increased risk of new‐onset AF. Our findings imply that treatment to reduce subclinical peripheral atherosclerosis might carry a potential for prevention of AF in the general population, especially among women, but future experimental studies are warranted to confirm our findings.

## Sources of Funding

The Rotterdam Study is funded by Erasmus Medical Center and Erasmus University, Rotterdam, Netherlands Organization for the Health Research and Development (ZonMw), the Research Institute for Diseases in the Elderly, the Ministry of Education, Culture and Science, the Ministry for Health, Welfare and Sports, the European Commission (DG XII), and the Municipality of Rotterdam. This study is further supported by the Gender and prevention grant (555003017) from ZonMw.

## Disclosures

Ikram reports consulting fees from BioGen Inc. The remaining authors have no disclosures to report.

## Supporting information

Data S1
Tables S1–S6
