# Supplementary material for: Subclinical Measures of Peripheral Atherosclerosis and the Risk of New‐Onset Atrial Fibrillation in the General Population: the Rotterdam Study
Source: J Am Heart Assoc. 2021 Dec 31;11(1):e023967. doi: 10.1161/JAHA.121.023967 (PMC9075211; doi:10.1161/JAHA.121.023967)
Supplement: Supplementary file 1 — Data S1 Tables S1–S6 [file JAH3-11-e023967-s001.pdf]

SUPPLEMENTAL MATERIAL

Data S1. Assessment of cardiovascular risk factors

Table S1. Association between baseline and longitudinal measures of carotid intima-media thickness and carotid plaque with the risk of new-onset atrial fibrillation in the total study population and stratified by sex with non-imputed data

Table S2. Association between baseline and longitudinal measures of ankle-brachial index with the risk of new-onset atrial fibrillation in the total study population and stratified by sex with non-imputed data

Table S3. Association between baseline and longitudinal measures of carotid intima-media thickness and carotid plaque with the risk of new-onset atrial fibrillation in the total study population and stratified by sex with exclusion of prevalent and incident coronary heart disease cases prior to incident atrial fibrillation

Table S4. Association between baseline and longitudinal measures of ankle-brachial index with the risk of new-onset atrial fibrillation in the total study population and stratified by sex with exclusion of prevalent and incident coronary heart disease cases prior to incident atrial fibrillation

Table S5. Association between baseline and longitudinal measures of carotid intima-media thickness and carotid plaque with the risk of mortality in the total study population and stratified by sex

Table S6. Association between baseline and longitudinal measures of ankle-brachial index with the risk of mortality in the total study population and stratified by sex

Data S1. Assessment of cardiovascular risk factors

All participants responded to comprehensive computerized questionnaires at baseline about their current health status, medical history, medication, and life style. They were interviewed at home by trained interviewers, and underwent more extensive clinical examination and laboratory assessments at the research center.

Standardized measurements of height (in cm) and weight (in kg) were performed and body mass index (BMI) was calculated as weight divided by height squared. Serum total and high-density lipoprotein (HDL) cholesterol were measured with an automated enzymatic method. Blood pressure was measured twice at the right upper arm with a random zero mercury sphygmomanometer in the sitting position. Systolic and diastolic blood pressures were calculated as the mean of the 2 consecutive measurements. Hypertension was defined as a systolic blood pressure of ≥140 mmHg or a diastolic blood pressure ≥90 mmHg or use of antihypertensive drugs prescribed for hypertension.(3,9) Smoking information derived from baseline questionnaires was categorized into never, former, and current smokers. Diabetes mellitus (DM) was defined as fasting serum glucose levels ≥7.0 mmol/L (126 mg/dL) (or non-fasting serum glucose levels ≥11.1 mmol/L (200 mg/dL) if fasting samples were unavailable) or the use of antidiabetic therapy. The assessment and definition of prevalent coronary heart disease (CHD) and heart failure (HF) has been described in detail previously.(15) Left ventricular hypertrophy (LVH) on the electrocardiogram (ECG) was diagnosed using the MEANS program with an algorithm that takes into accounts QRS voltages, with an age-dependent correction and repolarization. Medication use was derived from baseline questionnaires, pharmacy data, and was categorized and defined according to the World Health Organization Anatomical Therapeutic

Chemical (WHO ATC) classifications. Specifically, cardiac medication, antihypertensive medication, and lipid lowering medication were defined according to the WHO ATC categories c01, c02, and c10, respectively.

Table S1. Association between baseline and longitudinal measures of carotid intima-media thickness and carotid plaque with the risk of new-onset atrial fibrillation in the total study population and stratified by sex with non-imputed data

	Total study population		Men		Women	
	Cause-specific HR (95% CI)					
	Model 1 [*]	Model 2 [†]	Model 1 [*]	Model 2 [†]	Model 1 [*]	Model 2 [†]
**Cox proportional hazards models [‡]**						
**cIMT [‖]**	3.13 (2.01–4.88), $p=4.61 \times 10^{-07}$	2.02 (1.28–3.19), p=0.0026	1.99 (1.08–3.66), p=0.0273	1.26 (0.66–2.38), p= 0.4841	5.51 (2.88–10.56), $p=2.65 \times 10^{-07}$	3.77 (1.93–7.35), $p=9.70 \times 10^{-05}$
**Carotid plaque [‖]**	1.33 (1.15–1.54), p=0.0002	1.22 (1.05–1.42), p=0.0084	1.33 (1.07–1.64), p=0.0106	1.18 (0.95–1.48), p=0.1317	1.33 (1.09–1.62), p=0.0057	1.27 (1.03–1.56), p=0.0238
**cIMT, quartiles [‖]**						
**Q1 [¶]**	1 (ref)	1 (ref)	1 (ref)	1 (ref)	1 (ref)	1 (ref)
**Q2 [¶]**	1.14 (0.93–1.39), p=0.2166	1.06 (0.86–1.30), p=0.5904	1.23 (0.94–1.60), p=0.1395	1.15 (0.87–1.50), p=0.3284	1.26 (0.94–1.68), p=0.1257	1.15 (0.86–1.55), p=0.3390
**Q3 [¶]**	1.36 (1.11–1.65), p=0.0028	1.22 (1.00–1.50), p=0.0515	1.30 (0.99–1.71), p=0.0569	1.15 (0.87–1.52), p=0.3239	1.34 (1.00–1.79), p=0.0494	1.24 (0.93–1.67), p=0.1437

**Q4** [¶]	1.61 (1.31–1.96), $p=4.08\text{x}10^{-06}$	1.37 (1.11–1.68), p=0.0029	1.43 (1.08–1.89), p=0.0112	1.20 (0.91–1.60), p=0.2012	1.81 (1.35–2.42), $p=7.3\text{x}10^{-05}$	1.56 (1.16–2.09), p=0.0032
**Joint models** [§]						
**cIMT** [‖]	3.43 (1.98–5.63), p<0.0001	2.26 (1.39–3.75), p=0.0028	2.13 (1.06–4.10), p=0.0330	1.34 (0.65–2.68), p=0.4154	6.42 (3.11–12.95), p<0.0001	4.44 (2.02–9.31), p<0.0001
**Carotid plaque** [‖]	2.19 (1.49–3.46), p<0.0001	1.65 (1.13–2.47), p=0.0077	2.01 (1.18–3.73), p=0.0126	1.47 (0.89–2.68), p=0.1488	1.86 (1.18–3.10), p=0.0042	1.72 (1.07–2.93), p=0.0239

Abbreviations: CI, confidence interval; cIMT, carotid intima-media thickness; HR, hazard ratio; Q, quartiles.

[*] Adjusted for age, sex (if applicable), and cohort.

[†] Adjusted for age, sex (if applicable), cohort, body mass index, total cholesterol, high-density lipoprotein cholesterol, hypertension, smoking status, history of diabetes mellitus, history of coronary heart disease, history of heart failure, left ventricular hypertrophy on the electrocardiogram, use of cardiac medication, and use of lipid lowering medication.

Association between [‡] baseline carotid intima-media thickness and [§] longitudinal measures of carotid intima-media thickness, and carotid plaque for up to 3 repeated measurements during follow-up with incident atrial fibrillation, assessed by [‡] Cox proportional hazards models and [§] joint models.

[‖] Hazard ratios represent 1 unit increase in carotid intima-media thickness, and 1 unit increase in the probability of carotid plaque with the risk of new-onset atrial fibrillation.

[¶] Quartiles in the total study population were Q1: ≤0.72mm, Q2: 0.73–0.80mm, Q3: 0.81–0.90mm, Q4: ≥0.91mm.

Quartiles in men were Q1: ≤0.74mm, Q2: 0.75–0.83mm, Q3: 0.84–0.94mm, Q4: ≥0.95mm.

Quartiles in women were Q1: ≤0.70mm, Q2: 0.71–0.78mm, Q3: 0.79–0.88mm, Q4: ≥0.89mm.

Table S2. Association between baseline and longitudinal measures of ankle-brachial index with the risk of new-onset atrial fibrillation in the total study population and stratified by sex with non-imputed data

	**Total study population**		**Men**		**Women**	
	**Cause-specific HR (95% CI)**					
	**Model 1** *	**Model 2** †	**Model 1** *	**Model 2** †	**Model 1** *	**Model 2** †
**Cox proportional hazards models** ‡						
**ABI** ‖	2.18 (1.53–3.10), $p=1.62x10^{-05}$	1.66 (1.15–2.41), p=0.0071	2.71 (1.66–4.44), $p=7.06x10^{-05}$	1.97 (1.17–3.31), p=0.0107	1.73 (1.04–2.88), p=0.0337	1.41 (0.83–2.39), p=0.2084
**ABI, categories** ‖						
**≤0.90**	1.39 (1.16–1.66), p=0.0004	1.25 (1.04–1.51), p=0.0181	1.59 (1.22–2.06), p=0.0006	1.40 (1.07–1.83), p=0.0139	1.22 (0.95–1.57), p=0.1210	1.13 (0.87–1.46), p=0.3643
**0.91–0.99**	1.34 (1.10–1.63), p=0.0037	1.22 (1.00–1.48), p=0.0507	1.60 (1.20–2.13), p=0.0013	1.43 (1.07–1.91), p=0.0154	1.15 (0.88–1.51), p=0.2990	1.07 (0.81–1.40), p=0.6422
**1.00–1.40**	1 (ref)	1 (ref)	1 (ref)	1 (ref)	1 (ref)	1 (ref)
**Joint models** §						

**ABI** [‖]	8.91 (3.31–23.10),<br>p<0.0001	5.52 (1.93–15.69),<br>p=0.0014	9.48 (2.91–34.97),<br>p<0.0001	5.45 (1.67–18.88),<br>p=0.0098	7.03 (1.96–28.57),<br>p=0.0028	5.11 (0.97–22.93),<br>p=0.0533

Abbreviations: ABI, ankle-brachial index; CI, confidence interval; HR, hazard ratio.

[*] Adjusted for age, sex (if applicable), and cohort.

[†] Adjusted for age, sex (if applicable), cohort, body mass index, total cholesterol, high-density lipoprotein cholesterol, hypertension, smoking status, history of diabetes mellitus, history of coronary heart disease, history of heart failure, left ventricular hypertrophy on the electrocardiogram, use of cardiac medication, and use of lipid lowering medication.

Association between [‡] baseline ankle-brachial index and [§] longitudinal measures of ankle-brachial index for up to 2 repeated measurements during follow-up with incident atrial fibrillation, assessed by [‡] Cox proportional hazards models and [§] joint models.

[‖] Hazard ratios represent 1 unit decrease in ankle-brachial index with the risk of new-onset atrial fibrillation.

Table S3. Association between baseline and longitudinal measures of carotid intima-media thickness and carotid plaque with the risk of new-onset atrial fibrillation in the total study population and stratified by sex with exclusion of prevalent and incident coronary heart disease cases prior to incident atrial fibrillation

	**Total study population**		**Men**		**Women**	
	**Cause-specific HR (95% CI)**					
	**Model 1** *	**Model 2** †	**Model 1** *	**Model 2** †	**Model 1** *	**Model 2** †
**Cox proportional hazards models** ‡						
**cIMT** ‖	3.14 (1.99–4.95), $p=8.26x10^{–07}$	1.99 (1.24–3.18), p=0.0043	1.47 (0.73–2.95), p=0.2810	0.88 (0.42–1.83), p=0.7235	5.76 (3.17–10.46), $p=9.14x10^{–09}$	3.66 (1.99–6.76), $p=3.26x10^{–05}$
**Carotid plaque** ‖	1.25 (1.09–1.43), p=0.0013	1.17 (1.02–1.34), p=0.0259	1.16 (0.94–1.43), p=0.1680	1.08 (0.87–1.33), p=0.4995	1.31 (1.10–1.57), p=0.0026	1.23 (1.03–1.48), p=0.0224
**cIMT, quartiles** ‖						
**Q1** ¶	1 (ref)	1 (ref)	1 (ref)	1 (ref)	1 (ref)	1 (ref)
**Q2** ¶	1.20 (0.99–1.45), p=0.0641	1.12 (0.93–1.35), p=0.2466	1.23 (0.93–1.61), p=0.1417	1.13 (0.86–1.49), p=0.3962	1.19 (0.91–1.54), p=0.2000	1.10 (0.85–1.43), p=0.4575

**Q3** ¶	1.37 (1.14–1.66), p=0.0010	1.24 (1.03–1.51), p=0.0254	1.30 (0.99–1.71), p=0.0643	1.13 (0.85–1.50), p=0.4039	1.38 (1.07–1.79), p=0.0143	1.27 (0.98–1.64), p=0.0724
**Q4** ¶	1.64 (1.35–1.99), $p=8.97x10^{-07}$	1.37 (1.12–1.67), p=0.0021	1.30 (0.97–1.75), p=0.0775	1.07 (0.79–1.46), p=0.6545	1.81 (1.39–2.36), $p=1.08x10^{-05}$	1.53 (1.17–1.99), p=0.0018
**Joint models** §						
**cIMT** ‖	3.61 (2.22–5.83), p<0.0001	2.44 (1.52–3.99), p<0.0001	1.45 (0.67–3.20), p=0.3488	0.94 (0.41–2.08), p=0.8646	7.63 (4.03–14.89), p<0.0001	5.11 (2.52–10.05), p<0.0001
**Carotid plaque** ‖	1.83 (1.22–2.74), p=0.0028	1.62 (1.12–2.40), p=0.0084	1.51 (0.93–2.53), p=0.0933	1.26 (0.75–2.16), p=0.4084	1.99 (1.22–3.28), p=0.0035	1.78 (1.15–2.78), p=0.0105

Abbreviations: CI, confidence interval; cIMT, carotid intima-media thickness; HR, hazard ratio; Q, quartiles.

* Adjusted for age, sex (if applicable), and cohort.

† Adjusted for age, sex (if applicable), cohort, body mass index, total cholesterol, high-density lipoprotein cholesterol, hypertension, smoking status, history of diabetes mellitus, history of heart failure, left ventricular hypertrophy on the electrocardiogram, use of cardiac medication, and use of lipid lowering medication.

Association between ‡ baseline carotid intima-media thickness and § longitudinal measures of carotid intima-media thickness, and carotid plaque for up to 3 repeated measurements during follow-up with incident atrial fibrillation, assessed by ‡ Cox proportional hazards models and § joint models.

‖ Hazard ratios represent 1 unit increase in carotid intima-media thickness, and 1 unit increase in the probability of carotid plaque with the risk of new-onset atrial fibrillation.

¶ Quartiles in the total study population were Q1: ≤0.72mm, Q2: 0.73–0.80mm, Q3: 0.81–0.90mm, Q4: ≥0.91mm.

Quartiles in men were Q1: ≤0.74mm, Q2: 0.75–0.83mm, Q3: 0.84–0.94mm, Q4: ≥0.95mm.

Quartiles in women were Q1: ≤0.70mm, Q2: 0.71–0.78mm, Q3: 0.79–0.88mm, Q4: ≥0.89mm.

Table S4. Association between baseline and longitudinal measures of ankle brachial-index with the risk of new-onset atrial fibrillation in the total study population and stratified by sex with exclusion of prevalent and incident coronary heart disease cases prior to incident atrial fibrillation

	**Total study population**		**Men**		**Women**	
	**Cause-specific HR (95% CI)**					
	**Model 1 ***	**Model 2 †**	**Model 1 ***	**Model 2 †**	**Model 1 ***	**Model 2 †**
**Cox proportional hazards models ‡**						
**ABI ‖**	2.38 (1.68–3.36), $p=1.15\times10^{-06}$	1.83 (1.27–2.64), p=0.0012	2.82 (1.67–4.77), p=0.0001	2.24 (1.28–3.92), p=0.0049	2.08 (1.31–3.31), p=0.0020	1.62 (0.99–2.64), p=0.0531
**ABI, categories ‖**						
**≤0.90**	1.34 (1.12–1.61), p=0.0015	1.24 (1.03–1.49), p=0.0241	1.56 (1.17–2.07), p=0.0026	1.42 (1.06–1.92), p=0.0191	1.21 (0.96–1.53), p=0.1014	1.14 (0.90–1.44), p=0.2837
**0.91–0.99**	1.32 (1.09–1.60), p=0.0044	1.19 (0.98–1.44), p=0.0787	1.47 (1.07–2.02), p=0.0191	1.30 (0.94–1.80), p=0.1107	1.24 (0.98–1.57), p=0.0732	1.12 (0.88–1.42), p=0.3465
**1.00–1.40**	1 (ref)	1 (ref)	1 (ref)	1 (ref)	1 (ref)	1 (ref)

**Joint models** §						
**ABI** ‖	11.52 (4.11–31.96), p<0.0001	7.70 (2.38–25.71), p<0.0001	12.10 (3.43–40.05), p<0.0001	9.58 (2.59–38.28), p<0.0001	9.21 (2.79–29.16), p<0.0001	5.72 (1.58–22.81), p=0.0049

Abbreviations: ABI, ankle-brachial index; CI, confidence interval; HR, hazard ratio.

* Adjusted for age, sex (if applicable), and cohort.

† Adjusted for age, sex (if applicable), cohort, body mass index, total cholesterol, high-density lipoprotein cholesterol, hypertension, smoking status, history of diabetes mellitus, history of heart failure, left ventricular hypertrophy on the electrocardiogram, use of cardiac medication, and use of lipid lowering medication.

Association between ‡ baseline ankle-brachial index and § longitudinal measures of ankle-brachial index for up to 2 repeated measurements during follow-up with incident atrial fibrillation, assessed by ‡ Cox proportional hazards models and § joint models.

‖ Hazard ratios represent 1 unit decrease in ankle-brachial index with the risk of new-onset atrial fibrillation.

Table S5. Association between baseline and longitudinal measures of carotid intima-media thickness and carotid plaque with the risk of mortality in the total study population and stratified by sex

	**Total study population**		**Men**		**Women**	
	**Cause-specific HR (95% CI)**					
	**Model 1** [*]	**Model 2** [†]	**Model 1** [*]	**Model 2** [†]	**Model 1** [*]	**Model 2** [†]
**Cox proportional hazards models** [‡]						
**cIMT** [‖]	2.99 (2.39–3.75), $p<2.00x10^{-16}$	2.46 (1.96–3.09), $p=8.21x10^{-15}$	3.46 (2.52–4.76), $p=1.94x10^{-14}$	3.02 (2.17–4.20), $p=4.47x10^{-11}$	2.56 (1.86–3.51), $p=6.22x10^{-09}$	2.14 (1.56–2.93), $p=2.20x10^{-06}$
**Carotid plaque** [‖]	1.31 (1.22–1.41), $p=6.10x10^{-13}$	1.21 (1.13–1.31), $p=4.13x10^{-07}$	1.33 (1.18–1.49), $p=1.22x10^{-06}$	1.24 (1.10–1.39), p=0.0003	1.29 (1.17–1.42), $p=1.89x10^{-07}$	1.19 (1.08–1.31), p=0.0005
**cIMT, quartiles** [‖]						
**Q1** [¶]	1 (ref)	1 (ref)	1 (ref)	1 (ref)	1 (ref)	1 (ref)
**Q2** [¶]	1.00 (0.90–1.11), p=0.9914	0.98 (0.88–1.09), p=0.6890	1.04 (0.89–1.22), p=0.6088	1.01 (0.87–1.19), p=0.8578	0.98 (0.85–1.13), p=0.7694	0.97 (0.84–1.12), p=0.6771
**Q3** [¶]	1.22 (1.10–1.35), p=0.0002	1.19 (1.07–1.32), p=0.0009	1.33 (1.14–1.54), p=0.0002	1.28 (1.10–1.49), p=0.0014	1.13 (0.99–1.30), p=0.0804	1.14 (0.99–1.31), p=0.0747

**Q4** ¶	1.36 (1.22–1.50), $p=1.08x10^{-08}$	1.28 (1.15–1.42), $p=5.82x10^{-06}$	1.45 (1.25–1.69), $p=1.16x10^{-06}$	1.40 (1.19–1.63), $p=2.73x10^{-05}$	1.25 (1.08–1.44), p=0.0023	1.21 (1.05–1.39), p=0.0107
**Joint models** §						
**cIMT** ‖	2.78 (2.14–3.61), p<0.0001	2.25 (1.74–2.93), p<0.0001	3.15 (2.18–4.55), p<0.0001	2.66 (1.85–3.83), p<0.0001	2.52 (1.74–3.61), p<0.0001	2.13 (1.46–3.13), p<0.0001
**Carotid plaque** ‖	2.94 (2.19–4.10), p<0.0001	1.99 (1.50–2.71), p<0.0001	2.97 (1.93–4.92), p<0.0001	2.07 (1.36–3.34), p=0.0007	2.50 (1.72–3.80), p<0.0001	1.75 (1.23–2.58), p=0.0014

Abbreviations: CI, confidence interval; cIMT, carotid intima-media thickness; HR, hazard ratio; Q, quartiles.

[*] Adjusted for age, sex (if applicable), and cohort.

[†] Adjusted for age, sex (if applicable), cohort, body mass index, total cholesterol, high-density lipoprotein cholesterol, hypertension, smoking status, history of diabetes mellitus, history of coronary heart disease, history of heart failure, left ventricular hypertrophy on the electrocardiogram, use of cardiac medication, and use of lipid lowering medication.

Association between [‡] baseline carotid intima-media thickness and [§] longitudinal measures of carotid intima-media thickness, and carotid plaque for up to 3 repeated measurements during follow-up with mortality, assessed by [‡] Cox proportional hazards models and [§] joint models.

[‖] Hazard ratios represent 1 unit increase in carotid intima-media thickness, and 1 unit increase in the probability of carotid plaque with the risk of mortality.

[¶] Quartiles in the total study population were Q1: ≤0.72mm, Q2: 0.73–0.80mm, Q3: 0.81–0.90mm, Q4: ≥0.91mm.

Quartiles in men were Q1: ≤0.74mm, Q2: 0.75–0.83mm, Q3: 0.84–0.94mm, Q4: ≥0.95mm.

Quartiles in women were Q1: ≤0.70mm, Q2: 0.71–0.78mm, Q3: 0.79–0.88mm, Q4: ≥0.89mm.

Table S6. Association between baseline and longitudinal measures of ankle-brachial index with the risk of mortality in the total study population and stratified by sex

	**Total study population**		**Men**		**Women**	
	**Cause-specific HR (95% CI)**					
	**Model 1 [*]**	**Model 2 [†]**	**Model 1 [*]**	**Model 2 [†]**	**Model 1 [*]**	**Model 2 [†]**
**Cox proportional hazards models [‡]**						
**ABI [‖]**	3.25 (2.78–3.79), $p<2.00\text{x}10^{-16}$	2.63 (2.24–3.09), $p<2.00\text{x}10^{-16}$	3.66 (2.90–4.62), $p<2.00\text{x}10^{-16}$	2.76 (2.16–3.52), $p=3.64\text{x}10^{-16}$	2.90 (2.35–3.57), $p<2.00\text{x}10^{-16}$	2.51 (2.02–3.11), $p<2.00\text{x}10^{-16}$
**ABI, categories [‖]**						
**≤0.90**	1.64 (1.52–1.78), $p<2.00\text{x}10^{-16}$	1.49 (1.37–1.62), $p<2.00\text{x}10^{-16}$	1.73 (1.53–1.97), $p<2.00\text{x}10^{-16}$	1.51 (1.33–1.72), $p=4.94\text{x}10^{-10}$	1.57 (1.42–1.75), $p<2.00\text{x}10^{-16}$	1.47 (1.32–1.64), $p=2.13\text{x}10^{-12}$
**0.91–0.99**	1.28 (1.16–1.41), $p=7.73\text{x}10^{-07}$	1.22 (1.11–1.34), $p=7.35\text{x}10^{-05}$	1.28 (1.09–1.49), p=0.00195	1.19 (1.02–1.39), p=0.0287	1.27 (1.12–1.44), p=0.0002	1.25 (1.10–1.41), p=0.0005
**1.00–1.40**	1 (ref)	1 (ref)	1 (ref)	1 (ref)	1 (ref)	1 (ref)
**Joint models [§]**						

**ABI** ‖	22.79 (13.99–39.40), p<0.0001	15.67 (9.18–28.03), p<0.0001	17.26 (9.25–33.46), p<0.0001	10.46 (5.30–22.70), p<0.0001	22.89 (11.55–49.67), p<0.0001	17.95 (7.83–42.54), p<0.0001

Abbreviations: ABI, ankle-brachial index; CI, confidence interval; HR, hazard ratio.

[*] Adjusted for age, sex (if applicable), and cohort.

[†] Adjusted for age, sex (if applicable), cohort, body mass index, total cholesterol, high-density lipoprotein cholesterol, hypertension, smoking status, history of diabetes mellitus, history of coronary heart disease, history of heart failure, left ventricular hypertrophy on the electrocardiogram, use of cardiac medication, and use of lipid lowering medication.

Association between [‡] baseline ankle-brachial index and [§] longitudinal measures of ankle-brachial index for up to 2 repeated measurements during follow-up with mortality, assessed by [‡] Cox proportional hazards models and [§] joint models.

[‖] Hazard ratios represent 1 unit decrease in ankle-brachial index with the risk of mortality.
